# 

**Published:** 1908-10-15

**Authors:** 


### ANNOUNCEMENTS.

New Jersey Board of Examiners.—This Board will meet at Trenton, N. J., Monday, December 7th to 9th, in the State House. Candidates must furnish professional and preliminary credentials and a photograph of himself or his application will not be considered. Applications must also be filed by December 3rd. For full particulars address

Dr. C. A. Meeker,

29 Fulton Street,

Newark, N. J.

THE AMERICAN SOCIETY OF ORTHODONTISTS.—The eighth annual meeting of this societv will be held at Washingtcn, D. C., Monday, Tuesday and Wednesday, November 2, 3, and 4, 1908.

F. C. KEMPLE, *Secy.*,
43 W. 48th St.,
New York City.

—✦—

THE STATE DENTAL BOARD OF OHIO will meet in regular session in Columbus, cn October 21, 23, 1908, for the examination of applicants for license to practice dentistry in this State.

Only graduates of reputable dental colleges are eligible to appear for examination.

All applicaticns must be in the hands of the Secretary at least ten days before the date of the examinaticn, together with the fee cf $25.00.

For further information and blank application address the Secretary,

F. R. CHAPMAN,
305 Schultz Building,
Columbus, O.

—✦—

DEATH OF JOHN KERR—August seventeenth, John Kerr, the president of the Detroit Dental Manufacturing Co., died at his home in Detrcit. Mr. Kerr was a very active business man and had a fertile inventive genius, scme of the most useful specialties for making our work easier and more successful have come frcm his hands. He was a cordial and honorable gentleman, and all who knew him or ever dealt with him will be sorry to learn of his death.

—✦—

MASSACHUSETTS BOARD OF REGISTRATION IN DENTISTRY—A meeting of the Massachusetts Board of Registration in

Dentistry, for the examination of candidates, will be held in Boston, Mass., October 28, 29, and 30, 1908.

Candidates who have applied for examination will report to the secretary, Wednesday, October 28th, at 10 o'clock, A.M., at Tufts College Dental Infirmary, corner Rodgers and Huntington Avenues, prepared with rubber dam, gold, plastic filling materials and instruments, to demonstrate their skill in Operative Dentistry. Any one who wishes may bring his patient. So far as possible patients will be furnished. The Board in every instance selects the cavity to be filled. Partially prepared cavities never accepted.

The theoretic examination—written—will include operative dentistry, prosthetic dentistry, crown and bridge work, orthodontia, anatomy histology, surgery, pathology, materia medica, therapeutics, physiology, bacteriology, anesthesia, chemistry and metallurgy, and will be held at Civil Service Rooms, State House, from Thursday, October 29th, at 10:00 A.M., until Friday P.M., October 30th.

All applications, together with the fee of twenty dollars, if first examination, must be filed with the Secretary of the Board on or before October 21, 1908, as no application for this meeting will be received after that date.

*Candidates for second and subsequent examinations will be required to fill out an application blank (Form 2) and forward to the Secretary as above.*

Every candidate for examination must be twenty-one years of age.

Application blanks may be obtained from the Secretary.

Temporary licenses are never granted.

The fee for third and subsequent examinations is $5.00.

G. E. MITCHELL, D.D. S.,
*Secretary,*
Haverhill, Mass.

